# Supplementary material for: Metabolomic Profiling of Mice with Tacrolimus-Induced Nephrotoxicity: Carnitine Deficiency in Renal Tissue
Source: Biomedicines. 2024 Feb 26;12(3):521. doi: 10.3390/biomedicines12030521 (PMC10968022; doi:10.3390/biomedicines12030521)
Supplement: Supplementary file 1 [file biomedicines-12-00521-s001.zip › Figure S1.pdf]

Supplementary information for

Metabolomic Profiling of Mice with Tacrolimus-Induced Nephrotoxicity: Carnitine Deficiency in Renal Tissue

Sho Nishida [1,2], Tamaki Ishima [1], Natsuka Kimura [1], Daiki Iwami [2], Ryozo Nagai [3], Yasushi Imai [1] and Kenichi Aizawa [1,4,5,*]

[1] Division of Clinical Pharmacology, Department of Pharmacology, Jichi Medical University; Tochigi, Japan

[2] Division of Renal Surgery and Transplantation, Department of Urology, Jichi Medical University

[3] Jichi Medical University, Tochigi, Japan; rnagai@jichi.ac.jp

[4] Clinical Pharmacology Center, Jichi Medical University Hospital, Tochigi, Japan

[5] Division of Translational Research, Clinical Research Center, Jichi Medical University Hospital, Tochigi, Japan

* Correspondence: aizawa@jichi.ac.jp ; Tel.: +81(0)285-58-7388

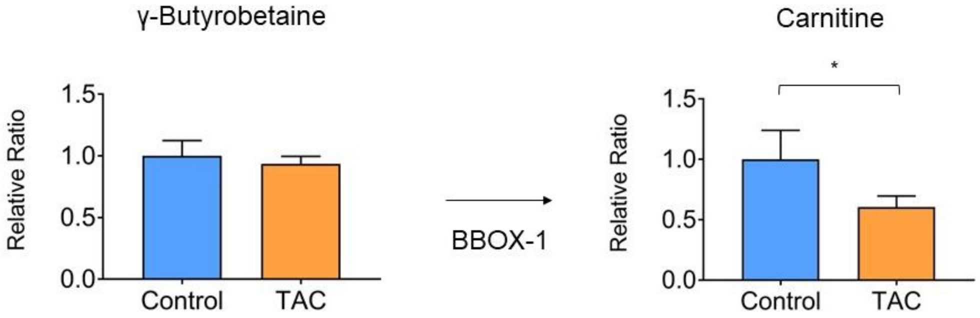

Supplemental Figure S1. **Relationship between γ-butyrobetaine and carnitine.** Solid line indicates the relationship between the substance and the produced metabolite. Carnitine is biosynthesized from γ-butyrobetaine via Gamma-butyrobetainenhydroxylase 1 (BBOX-1). The relative ratio of γ-butyrobetaine was 0.9 in the Tac group compared with the control group. G-butyrobetaine was not significantly different between the two groups (p = 0.37).
